# Combined Placement of Covered Self-Expanding Metallic Stents and Nasojejunal Tube for Managing Large Lower Esophageal Perforations

**DOI:** 10.14740/gr593w

**Published:** 2014-03-14

**Authors:** Surinder S Rana, Rajesh Gupta, Divya Dahiya, Arunanshu Behera, Deepak K Bhasin

**Affiliations:** a Department of Gastroenterology, Post Graduate Institute of Medical Education and Research (PGIMER), Sector 12, Chandigarh 160012, India; b Department of Surgery, Post Graduate Institute of Medical Education and Research (PGIMER), Sector 12, Chandigarh 160012, India

**Keywords:** Esophagus, Stents, Leaks, Bariatric surgery

## Abstract

Covered self-expanding metallic stents (cSEMSs) have emerged as effective treatment option for esophageal perforations. However, the large lower esophageal perforations where the cSEMS is placed across gastroesophageal junction have lower healing rates because refluxed gastric contents constantly irritate perforation and also there is increased risk of stent migration. Moreover, gastric mucosa tends to prolapse into lumen of lower end of stent causing its obstruction, leading to seepage of saliva and fluids from upper end of stent even in the patients who are on parenteral nutrition. We present our experience of a novel technique of combined cSEMS and nasojejunal tube (NJT) placement in four patients (two males) with benign large lower esophageal perforations. The NJT was placed through the stent into the jejunum through which patients were given enteral feeding. The stents were placed 5 - 21 days after esophageal perforation with the size of perforation ranging from 4 to 6 cm. As the NJT formed a loop in stomach, it prevented migration of stent. And also its presence in lumen of stent prevented its obstruction by prolapsing gastric mucosa, thereby preventing seepage of saliva and fluids from side of stent. Both stents and NJT were removed after 6 weeks and leak closed in all patients. Combined cSEMS and NJT placement seems to be safe and effective for treating large lower esophageal perforations. NJT placement seems to decrease risk of migration, prevents seepage of fluids and permits early enteral nutrition, thereby improving the healing rates.

## Introduction

Esophageal perforations and leaks are associated with high morbidity and mortality [1-3]. Their management is complex and involves treatment of sepsis and nutritional management along with definitive management of the esophageal defect. Surgery has been the mainstay of treatment of esophageal leaks with various procedures like resection, repair, patching and esophageal diversion being the commonly used procedures [2-4]. However, surgery has been associated with increased morbidity and mortality.

Recently, temporary endoscopic placement of fully covered self-expanding metallic stents (FcSEMSs) or partially covered self-expanding metallic stents (PcSEMSs) or self-expanding plastic stents (SEPSs) have emerged as safe and effective minimally invasive treatment option for esophageal perforations [1-3, 5-7]. The stents by effectively sealing the esophageal defect protect the healing mucosa from secretions and thus lead to the closure of the defect. However, the large lower esophageal perforations where the cSEMS is placed across gastroesophageal junction (GEJ) have lower healing rates because refluxed gastric contents constantly irritate perforation and there is also increased risk of stent migration [8, 9]. One recent comparative study has shown that endoscopic stent insertion in patients with Boerhaave syndrome offers no advantage regarding morbidity, intensive care unit or hospital stay, and is associated with frequent treatment failure eventually requiring surgical intervention and higher mortality than primary surgical therapy [9]. Moreover, gastric mucosa tends to prolapse into lumen of lower end of stent causing its obstruction, leading to seepage of saliva and fluids from upper end of stent even in the patients who are on parenteral nutrition thus causing failure of stent therapy.

In this case series, we present our successful experience of a novel technique of combined FcSEMS and nasojejunal tube (NJT) placement in four patients with benign large lower esophageal perforations.

## Case Report

### Case 1

**Figure 1:**
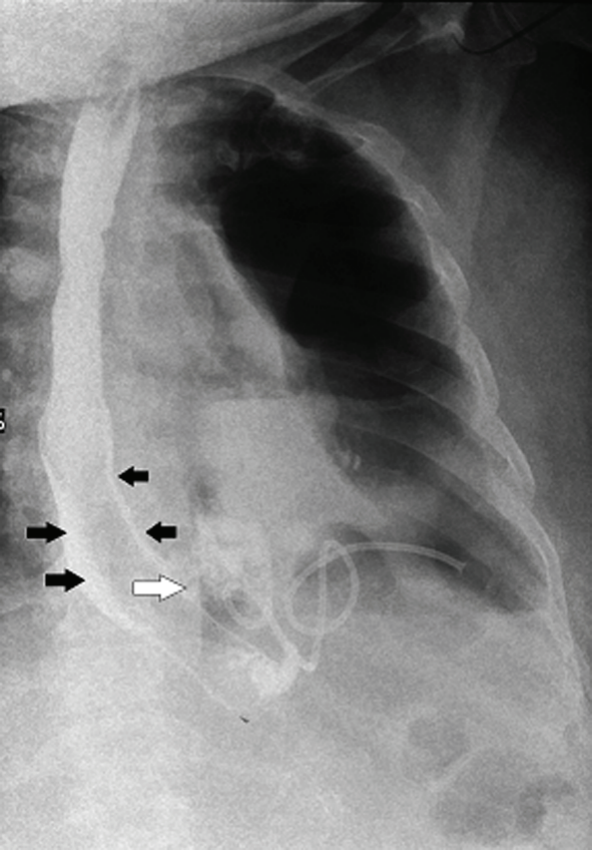
Contrast study of esophagus: no contrast is seen going across the lower end of stent. Contrast is seen seeping along the sides of the SEMS (black arrow) and then leaking into pleural cavity (white arrow). A pigtail is seen inside the left pleural cavity.

**Figure 2:**
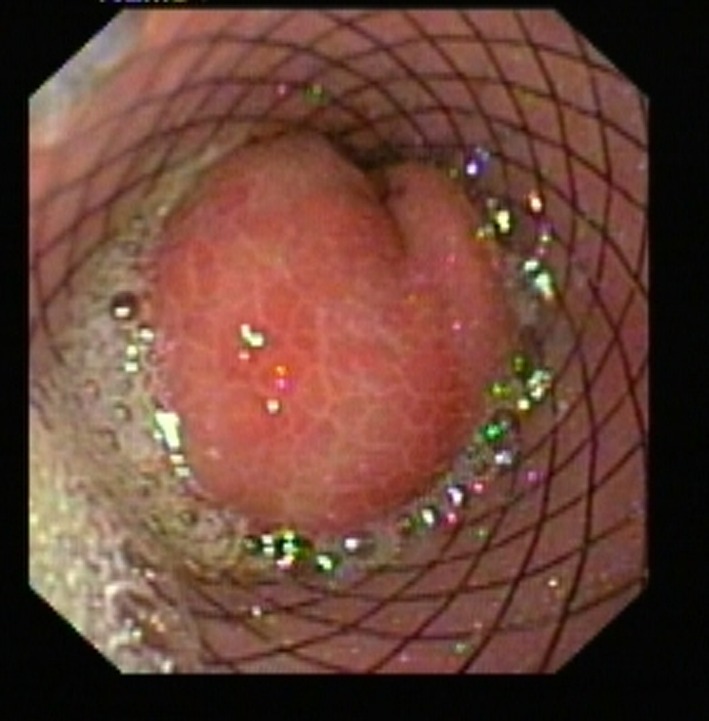
Lower end of SEMS blocked by prolapsed gastric mucosa.

**Figure 3:**
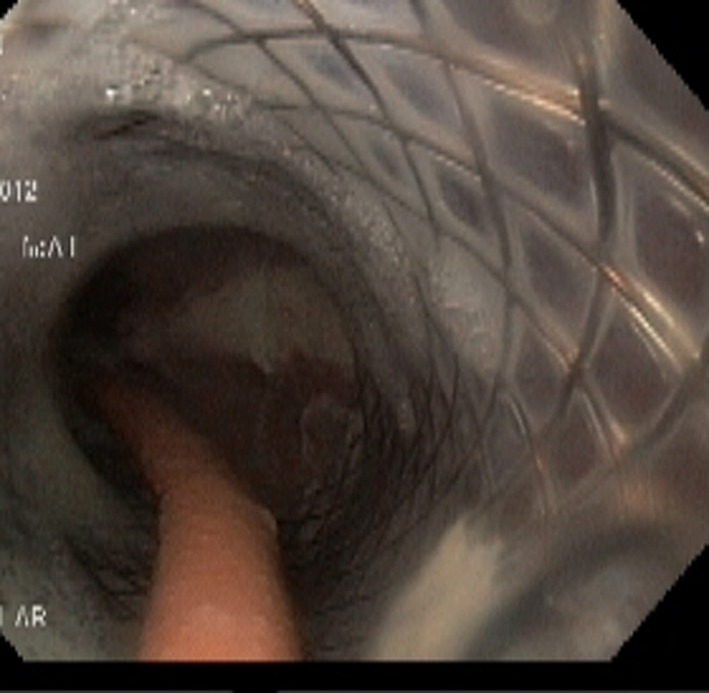
Endoscopy done 3 weeks after placement of NJT. The NJT is seen going through the SEMS and lower end of SEMS is seen opened up.

A 58-year-old male presented with fever and breathlessness 10 days after laparoscopic sleeve gastrectomy (LSG). Investigations revealed leukocytosis with left-sided hydropneumothorax. An emergent pigtail insertion into pleural cavity was done and frank pus was drained. The esophageal contrast study revealed a large lower esophageal perforation with contrast leaking into the left pleural cavity. An upper gastrointestinal endoscopy revealed a large defect in the lower esophagus measuring about 4 cm in length. The patient was started on intravenous antibiotics and after an informed consent, an FcSEMS (SX-Ella, Ella CS, Czech Republic; stent body diameter 20 mm and throats diameter of 25 mm with length of 11 cm) was placed across the GEJ. The patient was kept nil orally and parenteral nutrition was given. In spite of these measures, the fever persisted and the drain output was > 200 mL/day. The chest X-ray done on the fourth day revealed distal migration of the stent into the stomach. Using grasping forceps, the stent was repositioned into the esophagus. There was no decrease in the daily drain output and a repeat contrast study was done on the fourth day after repositioning of the stent. There was no passage of contrast across the distal end of the stent and because of this obstruction, it was found to be seeping through the upper end of the stent into the leak (Fig. 1). An endoscopy revealed the blockage of the lower end of the stent by the prolapsed gastric mucosa (Fig. 2). The prolapsed gastric mucosa was pushed by the endoscope and an NJT was placed through the SEMS (Fig. 3). Following this patient had gradual improvement with decreasing daily drain output and he was started on enteral feeding through the NJ tube. The contrast study done on the sixth day of NJT placement revealed free passage of the contrast into the residual stomach with no leakage. The pigtail was removed 4 weeks later and the stent was removed 6 weeks after insertion using the Ella extractor. Endoscopy revealed complete healing of the esophageal defect and there was no leakage on contrast esophagogram.

### Case 2

**Figure 4:**
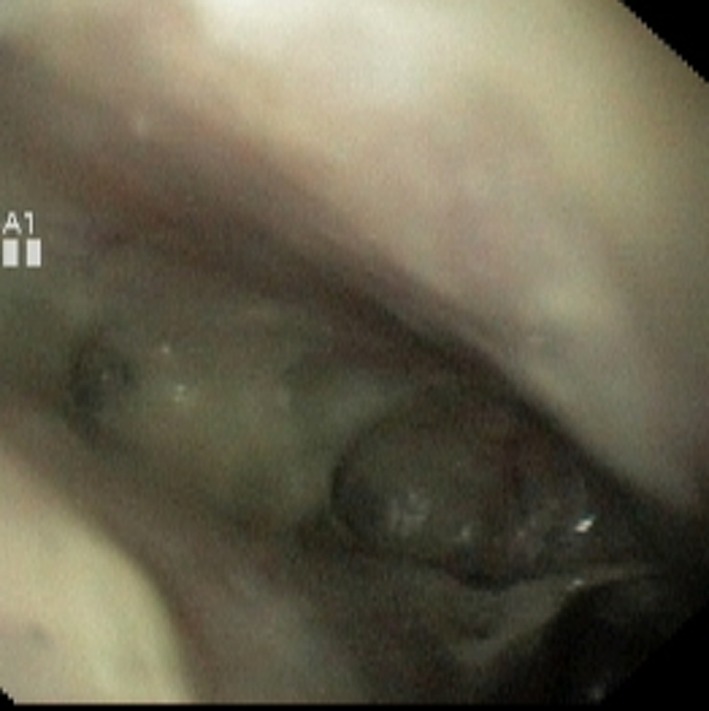
Large perforation at lower end of esophagus.

**Figure 5:**
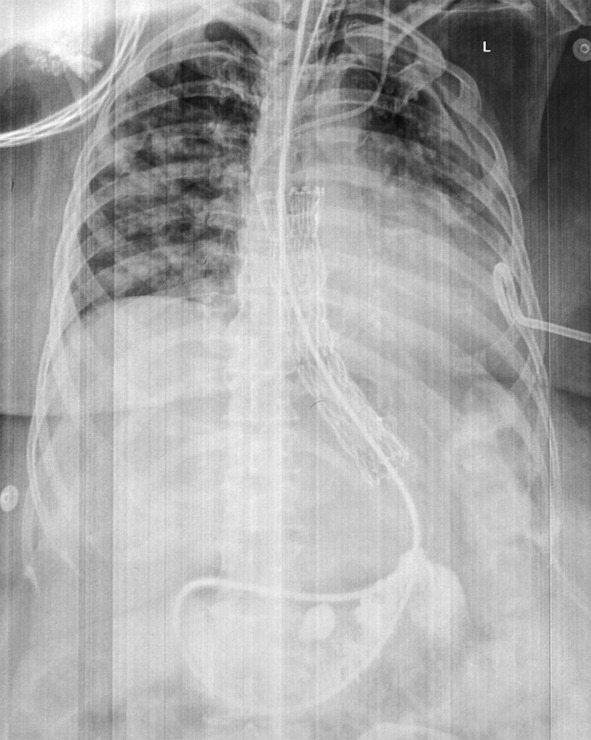
NJT is seen passing through SEMS into the jejunum. Pigtail in left pleural cavity and central line catheter are also noted.

A 48-year-old female presented with fever and breathlessness 5 days after LSG. Investigations revealed leukocytosis with left-sided hydropneumothorax. An emergent pigtail insertion was done and frank pus was drained. An upper gastrointestinal endoscopy revealed a large defect in the lower esophagus measuring about 6 cm in length through which the cardiac pulsations could be seen (Fig. 4). The patient was started on intravenous antibiotics and after an informed consent, an FcSEMS with anti-reflux valve (SX-Ella stent of length of 11 cm) was placed across the GEJ. Post stent placement, patient had atrial fibrillation with fast ventricular rate that was reverted back to sinus rhythm using intravenous amiodarone. Twenty-four hours later, the stent was found to be migrated into the stomach. The stent was pulled back and an NJT was placed through the stent and she was given enteral feeding through the NJT (Fig. 5). The contrast study done on the fourth day of NJT placement revealed free passage of the contrast into the residual stomach with no leakage. The pigtail was removed 5 weeks later and the stent was removed 6 weeks after insertion using the grasping forceps. Endoscopy revealed complete healing of the esophageal defect.

### Case 3

A 34-year-old female presented with fever and breathlessness 9 days after LSG. An emergent tube thoracostomy was done and frank pus was drained. The esophageal contrast study revealed a large lower esophageal perforation with contrast leaking into the left pleural cavity. The patient was started on intravenous antibiotics and after an informed consent, an FcSEMS (SX-Ella stent of length of 11 cm) was placed across the GEJ and NJT was placed through the stent into the jejunum through which she was given enteral feeding. The patient had gradual improvement and chest tube was removed 3 weeks later and stent was removed 6 weeks after insertion.

### Case 4

**Figure 6:**
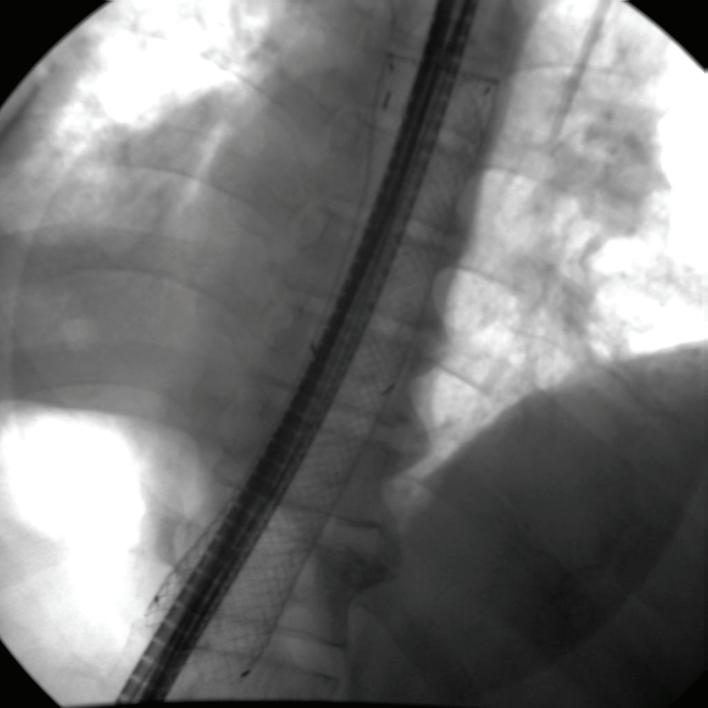
NJT placed through the SEMS.

**Figure 7:**
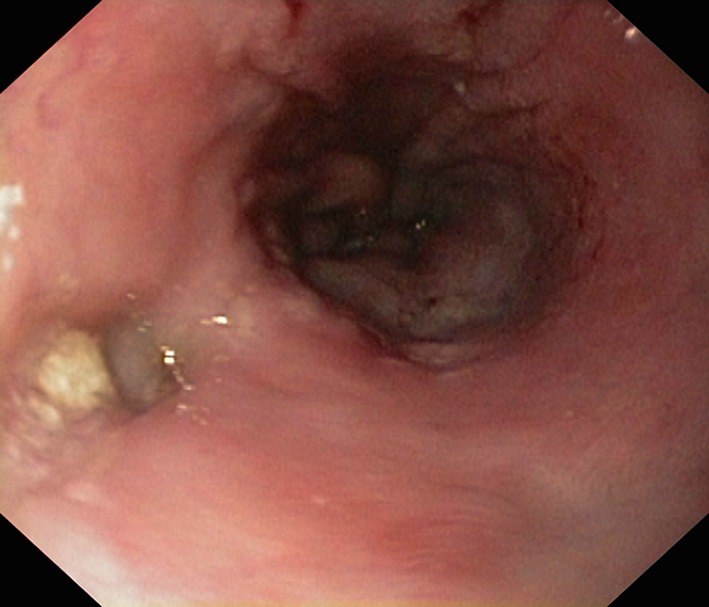
Endoscopy after stent removal: small depression is noted at the site of perforation.

**Figure 8:**
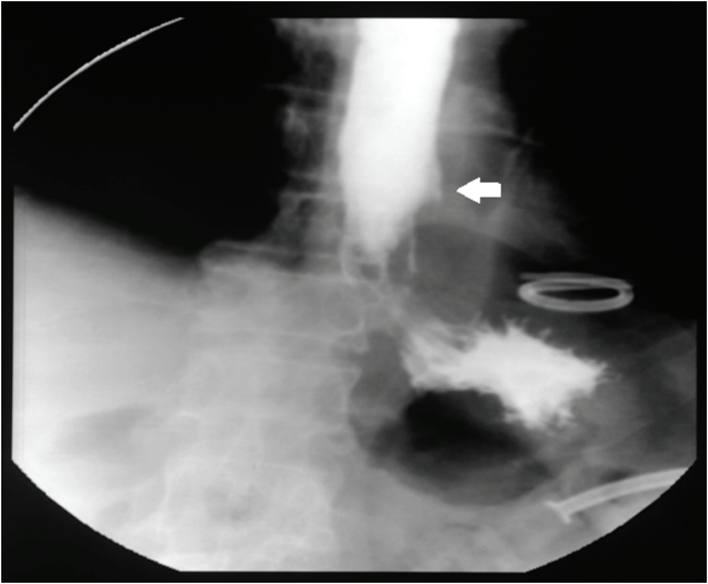
Contrast study after stent removal: no leakage of contrast is seen and a small outpouching is seen at site of perforation (arrow).

A 48-year-old male presented to another center with chest pain and breathlessness following repeated bouts of vomiting. On evaluation the patient was found to be having left-sided hydropneumothorax. An emergent tube thoracostomy was done and frank pus was drained. Computed tomography of the chest revealed leakage from the lower end of esophagus. He was treated with intravenous antibiotics and parenteral nutrition. As there was no improvement, he was referred to our center on the 21st day. The esophageal contrast study revealed a large lower esophageal perforation. After an informed consent, an FcSEMS with anti-reflux valve (SX-Ella stent of length of 11 cm) was placed across the GEJ and NJT was placed through the stent into the jejunum through which he was given enteral feeding (Fig. 6). The contrast study done on the fourth day of NJT placement revealed free passage of the contrast into stomach with no leakage. The chest tube was removed 5 weeks later, pigtail inserted into a loculated pleural collection and the stent was removed 6 weeks after insertion using the Ella extractor. Endoscopy performed after stent extraction revealed a small depression at the site of perforation (Fig. 7) and contrast study did not reveal any leakage of contrast and a small outpouching was seen at site of perforation (Fig. 8). Following this the pigtail was also removed.

## Discussion

Although temporary endoscopic placement of FcSEMS has been shown to be safe and effective minimally invasive treatment option for esophageal perforations but their results in large lower esophageal perforations where the cSEMS is placed across the GEJ are not so encouraging [8, 9]. One of the reasons for lower healing rates is the constant irritation of the perforation site by the refluxed gastric contents [8]. Increased migration rate of FcSEMS is also of concern. And, as shown in the first case, the gastric mucosa tends to prolapse into lumen of lower end of stent causing its obstruction, leading to seepage of saliva and fluids from upper end of stent and causing failure of stent therapy.

In the current case series, we have shown that placement of NJT through the FcSEMS improves the healing rates in large lower esophageal perforations. The placement of NJT has following advantages:

1) As the NJT formed a loop in the stomach, this contour of NJT seemed to help in anchorage of the stent and thus prevented migration of SEMS as shown in all four cases.

2) And also its presence in lumen of stent prevented its obstruction at the lower end by prolapsing gastric mucosa.

3) Enteral nutrition could be given through NJT without fear of seepage from the side of the SEMS and thus obviating costs and side effects of parenteral nutrition.

The long-term insertion of NJT is associated with discomfort to the patient and there is risk of pulling it out also. However, in our series all the patients tolerated the NJT well and in none of the patients it was pulled out.

In conclusion, combined cSEMS and NJT placement seems to be safe and effective for treating large lower esophageal perforations. NJT placement seems to decrease risk of migration, prevents seepage of fluids and permits early enteral nutrition, thereby improving the healing rates.

## References

[1] Adamek HE, Jakobs R, Dorlars D, Martin WR, Kromer MU, Riemann JF (1997). Management of esophageal perforations after therapeutic upper gastrointestinal endoscopy. Scand J Gastroenterol.

[2] Jougon J, Delcambre F, MacBride T, Minniti A, Velly JF (2002). [Mortality from iatrogenic esophageal perforations is high: experience of 54 treated cases]. Ann Chir.

[3] Swinnen J, Eisendrath P, Rigaux J, Kahegeshe L, Lemmers A, Le Moine O, Deviere J (2011). Self-expandable metal stents for the treatment of benign upper GI leaks and perforations. Gastrointest Endosc.

[4] Bufkin BL, Miller JI, Mansour KA (1996). Esophageal perforation: emphasis on management. Ann Thorac Surg.

[5] Freeman RK, Ascioti AJ, Wozniak TC (2007). Postoperative esophageal leak management with the Polyflex esophageal stent. J Thorac Cardiovasc Surg.

[6] van Boeckel PG, Dua KS, Weusten BL, Schmits RJ, Surapaneni N, Timmer R, Vleggaar FP (2012). Fully covered self-expandable metal stents (SEMS), partially covered SEMS and self-expandable plastic stents for the treatment of benign esophageal ruptures and anastomotic leaks. BMC Gastroenterol.

[7] van Boeckel PG, Sijbring A, Vleggaar FP, Siersema PD (2011). Systematic review: temporary stent placement for benign rupture or anastomotic leak of the oesophagus. Aliment Pharmacol Ther.

[8] David EA, Kim MP, Blackmon SH (2011). Esophageal salvage with removable covered self-expanding metal stents in the setting of intrathoracic esophageal leakage. Am J Surg.

[9] Schweigert M, Beattie R, Solymosi N, Booth K, Dubecz A, Muir A, Moskorz K (2013). Endoscopic stent insertion versus primary operative management for spontaneous rupture of the esophagus (Boerhaave syndrome): an international study comparing the outcome. Am Surg.

